# Comparison of the performance of digital variance angiography and digital subtraction angiography in children with arteriovenous malformations: a retrospective observational study

**DOI:** 10.1186/s41747-025-00614-w

**Published:** 2025-08-12

**Authors:** Balázs Bence Nyárády, Renáta Gubán, Ákos Pataki, András Bibok, Zsuzsanna Mihály, Dávid Korda, Dénes Horváthy, Anikó Ilona Nagy, János Pál Kiss, Edit Dósa

**Affiliations:** 1 Heart and Vascular Center, Semmelweis University, Budapest, Hungary; 2 Kinepict Health Ltd, Budapest, Hungary

**Keywords:** Angiography (digital subtraction), Arteriovenous malformations, Child, Contrast media, Radiation protection

## Abstract

**Background:**

Reducing contrast agent and radiation exposure is paramount for pediatric patients. Digital variance angiography (DVA) might address this need by increasing the contrast-to-noise ratio (CNR).

**Materials and methods:**

A total of 132 raw iodinated contrast angiograms of 10 children (mean age: 12 years) who had endovascular procedures for arteriovenous malformations were retrospectively processed for DVA analysis. The CNR of the DVA and digital subtraction angiography (DSA) images was calculated. The visual image quality was assessed using a four-point Likert scale. Statistical analyses were based on the Wilcoxon signed-rank test and one-sample *t*-test.

**Results:**

The CNR was determined and compared for 3,318 regions of interest in 132 image pairs in four anatomical regions (upper limb (UL), lower limb (LL), head and neck (HN), and chest (CH)). DVA outperformed DSA, with a median overall CNR_DVA_/CNR_DSA_ ratio of 2.00 (UL, 1.83; LL, 1.71; HN, 2.06; CH, 2.23; all *p* < 0.001). The paired Likert scale scores were significantly different from zero in 50% of the comparisons (in all large vessel and small vessel groups, except in the UL region, and the tissue blush group in the LL and HN regions), indicating a superiority of DSA, but the difference was clinically negligible.

**Conclusion:**

Although DVA improved CNR, it did not surpass DSA in subjective image quality, possibly due to motion artifacts and the high baseline quality of DSA images.

**Relevance statement:**

The enhanced CNR seen with DVA indicates a potential quality reserve that could be exploited to safely reduce contrast agent dose and radiation risks in pediatric patients, who are more susceptible to the long-term effects of radiation.

**Key points:**

- In previous studies, DVA was superior to DSA due to a higher CNR and better image quality. However, no evidence was available regarding pediatric endovascular procedures.
- While DVA exhibited a marked advantage in terms of the CNR, it was unable to surpass DSA in terms of visual assessment.
- The enhanced CNR seen with DVA indicates a potential quality reserve that could be exploited to safely reduce contrast agent dose and radiation risks in pediatric patients.

**Graphical Abstract:**

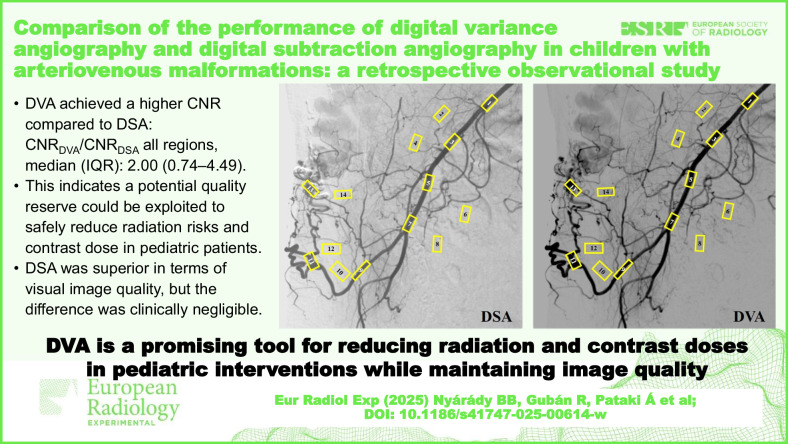

## Background

Congenital vascular malformations are a subset of vascular anomalies typically diagnosed in the first two decades of life, affecting approximately 0.5% of the European population [1]. In 1996, the International Society for the Study of Vascular Anomalies established a comprehensive classification system for vascular anomalies, which was revised in 2018 [1, 2]. Arteriovenous malformations (AVMs) are a prevalent subtype of vascular malformations. The above classification defines AVMs as high-flow vascular anomalies [1, 2]. Catheter-directed angiography is essential for planning and performing invasive treatment of AVMs [3, 4]. Pediatric AVMs present a unique clinical challenge due to the complexity of the lesions and the long-term radiation risks, as reducing the size or preventing the growth of AVMs is usually achieved by multiple radiological interventions rather than a single one.

The conventional method of catheter-directed angiography involves administering an iodinated contrast agent, either intra-arterially or intravenously, to visualize blood vessels. Meticulous removal of the radiopaque structures from the images ensures an accurate assessment of blood vessels. The resultant images are digital subtraction angiography (DSA) images. Notably, iodinated contrast agents are potentially toxic, particularly in patients with impaired renal function. Moreover, ionizing radiation exposure has non-negligible adverse effects on the patient (especially in younger age groups) and the personnel conducting the procedure [5–7].

A substantial body of research is underway to determine the optimal approach for endovascular interventions, aiming to minimize the use of contrast agents and reduce radiation exposure, while preserving image quality. Digital variance angiography (DVA) is a relatively novel technology based on the principles of kinetic imaging. It derives data from images obtained with penetrating radiation [8, 9]. Contrary to the DSA method, the DVA approach does not utilize a mask for subtraction. Instead, it calculates the standard deviation of the x-ray attenuation of each pixel. This processing algorithm extracts more information from the raw, unsubtracted acquisitions than the DSA method. Additionally, it improves image quality by amplifying the signal of the moving (flowing) contrast agent while suppressing background noise [10–16].

It has been shown that the superior quality of DVA can be used effectively to reduce the amount of contrast agent [17] or the radiation dose [18, 19]. This dose management capability would greatly benefit angiography in pediatric patients. However, the qualitative and quantitative indicators of DVA images have not yet been compared with those of DSA images in children, in whom catheter-directed diagnostic and/or therapeutic procedures are routinely performed with less contrast agent and reduced radiation doses. Therefore, this retrospective study aimed to investigate the performance of DVA in pediatric patients. AVMs were chosen as the model condition for this study because in these lesions, it is often possible to evaluate different types of vascular structures, tissue blush, and venous outflow simultaneously.

## Methods

The study was carried out following the ethical standards outlined in the 1964 Helsinki Declaration [20] and the regulations set by the national research committee. The study was approved by the Semmelweis University Regional and Institutional Committee of Science and Research Ethics (approval number 182/2022). Prior to access, all data were fully anonymized, and the aforementioned ethics committee waived the requirement for informed consent for the study. This retrospective observational study analyzed 10 patients (mean age, 12 years (range, 7–17 years), four males and six females) with a solitary AVM who underwent 15 endovascular interventions between December 2022 and December 2024 at the Heart and Vascular Center of Semmelweis University.

### DSA and DVA image generation

Before the diagnostic or therapeutic DSA examination, the interventional radiologist explained the procedure and its possible complications in detail to the patient (if the patient was at least of school age) and the parents, and obtained the parents’ verbal and written consent. The endovascular procedures were executed by two interventional radiologists (Á.P. and E.D.), each with over 20 years of experience. The volume and rate of contrast agent administration were tailored to the patient and the lesion, ranging from 15 to 147 mL per intervention. The intra-arterial contrast agents utilized included Ultravist (370 mg I/mL; Bayer AG), Iomeron (300 mg I/mL; Bracco Imaging SpA), and Omnipaque (300 mg I/mL; GE HealthCare Technologies Inc.). The acquisition of raw angiography images was performed at a rate of two or four frames per second using a Siemens Artis zee angiography machine (Siemens Healthineers AG) with a 30 × 40 cm detector. DSA and DVA images were derived from the same raw angiography image series for the study. DSA images were created on the Syngo workstation (Siemens Healthineers AG), while the DVA images were produced using the Kinepict Medical Imaging Tool v5.3 (Kinepict Health Ltd, Budapest, Hungary). Generating DSA and DVA images involved postprocessing steps, such as motion correction (pixel shift) and brightness/contrast adjustment, performed by a dedicated interventional radiologist (E.D.) using Syngo (for DSA images) and Kinepict software (for DVA images). Therefore, there was no discernible difference between the two image types in this respect. The calculated images were then employed to determine the contrast-to-noise ratio (CNR) and web-based visual evaluation.

### Image analysis: CNR

**Fig. 1:**
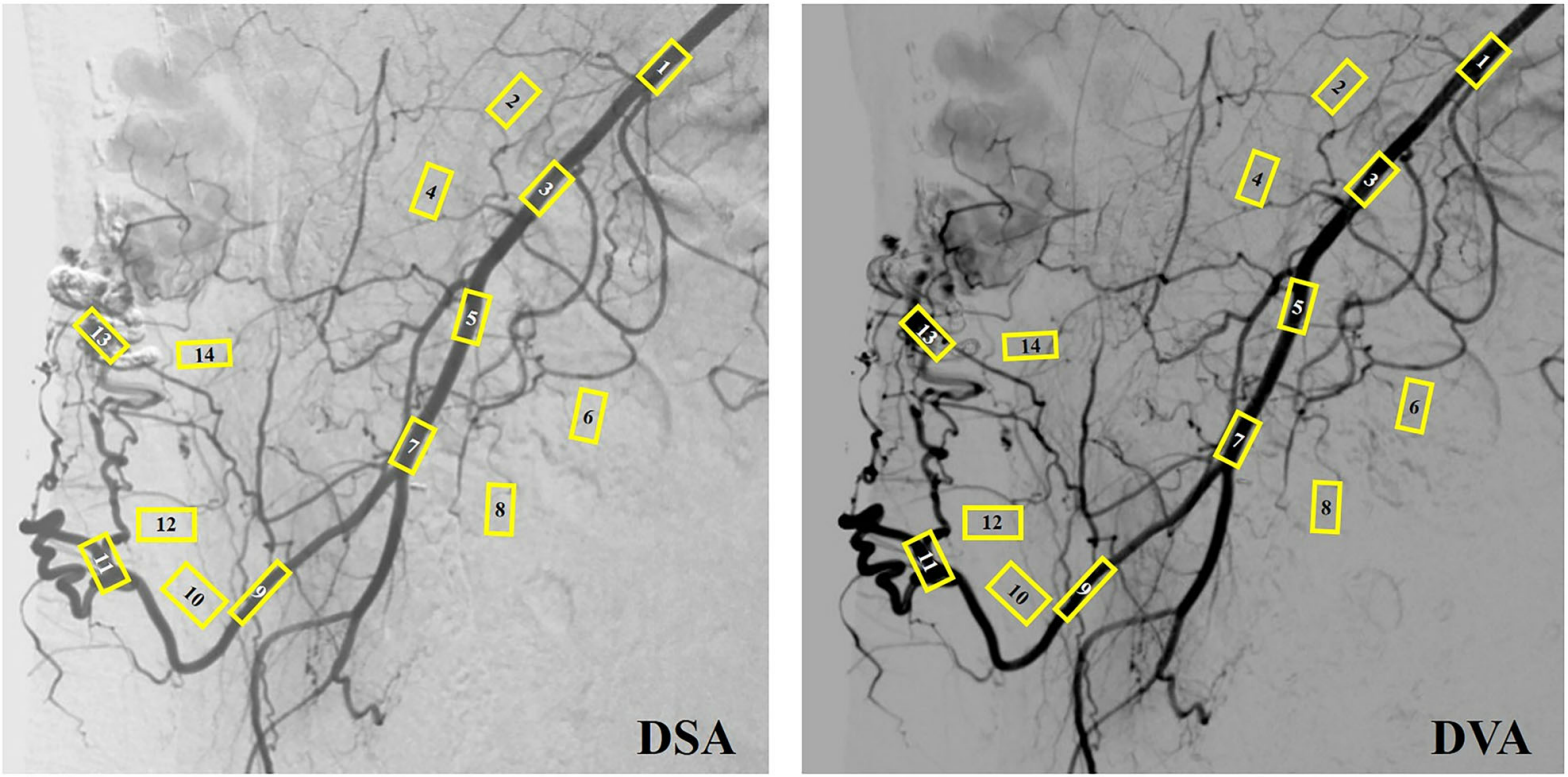
Contrast-to-noise ratio comparison of digital subtraction angiography and digital variance angiography images in a chest wall arteriovenous malformation. DSA, Digital subtraction angiography; DVA, Digital variance angiography

To obtain the CNR, regions of interest (ROIs) were manually selected on the AVM and the background. Then, pairs of ROIs were formed, consisting of a vascular ROI (placed on a contrast-filled vessel or blush) and an adjacent background ROI (placed on soft tissue or an unenhanced area). On average, 25 ROI pairs were defined for each AVM (Fig. 1). When a geometric discrepancy arose between the DSA and DVA images due to pixel shift, the ROIs of the DVA image were aligned with the ROIs of the corresponding DSA image. The calculation of the CNR for each ROI pair was completed using the following formula:

$${CNR}=\frac{({{Mean}}_{v}-{{Mean}}_{b})}{{{SD}}_{b}}$$

where Mean_v_ and Mean_b_ refer to the mean pixel intensity value of the vascular (Mean_v_) and background (Mean_b_) ROIs, while SD_b_ refers to the standard deviation value of the pixel intensity of the background ROIs. The CNR was subsequently computed for both DSA and DVA ROI pairs. The ratio of the CNR of DVA to the CNR of DSA (CNR_DVA_/CNR_DSA_) was also determined. ROIs were identified using Fiji software (version 2.0.0-rc-68/1.52e; National Institutes of Health).

### Image analysis: quality assessment

A web-based survey was conducted in a randomized and blinded manner, with DSA and DVA images evaluated by four interventional radiologists (Á.P., A.B., D.K., and D.H.) and a vascular surgeon (Z.M.) with a minimum of five years of experience. The images were compared using a four-point Likert scale, with the visibility and diagnostic value of large vessels, small vessels, tissue blush (if applicable), and the venous phase (if present) considered. The images encompassed four anatomical regions: upper extremities (14 image pairs), lower extremities (56 image pairs), head and neck (23 image pairs), and chest (39 image pairs). The image pairs were graded as follows: 0 = same, 1 = slightly better, 2 = clearly better, and 3 = better in all respects. The image pairs were presented randomly, without revealing the image type. Each image pair was rated on a single occasion by each reader, and all five experts compared all image pairs.

### Statistical analysis

The Stata 15.0 (StataCorp LLC) and GraphPad Prism 8.4.2 (GraphPad Software Inc.) programs were used for statistical analysis. The CNR values were expressed as median and interquartile range, and a comparison was made using the Wilcoxon signed-rank test. For the visual assessment scores, the mean and standard error of the mean were determined. The standard error of the mean was used instead of the standard deviation, as the primary goal was not to describe the variability of individual scores, but to report the reliability of the mean estimate. The deviation from 0, representing an equal quality level, was analyzed by the one-sample *t*-test. The normality of the distribution was investigated using the Kolmogorov–Smirnov test. Kendall’s *W* was calculated to test for agreement among observers, with possible values of 0 (no agreement), 0.1 (weak agreement), 0.3 (moderate agreement), 0.6 (strong agreement), and 1 (perfect agreement). The sample size (*n* = 132 image pairs) was determined based on available data; a post hoc power analysis confirmed > 95% power to detect medium effect sizes (*r* = 0.3) at a two-sided alpha of 0.05. Statistical significance was defined as *p* < 0.05.

## Results

The patients had no known comorbidities and were not taking any regular medications. Two AVMs were identified in the upper limb, four in the lower limb, two in the head and neck region, and two in the chest. Three of the 15 endovascular procedures performed were diagnostic, while 12 were therapeutic.

### Image analysis: CNR

**Fig. 2:**
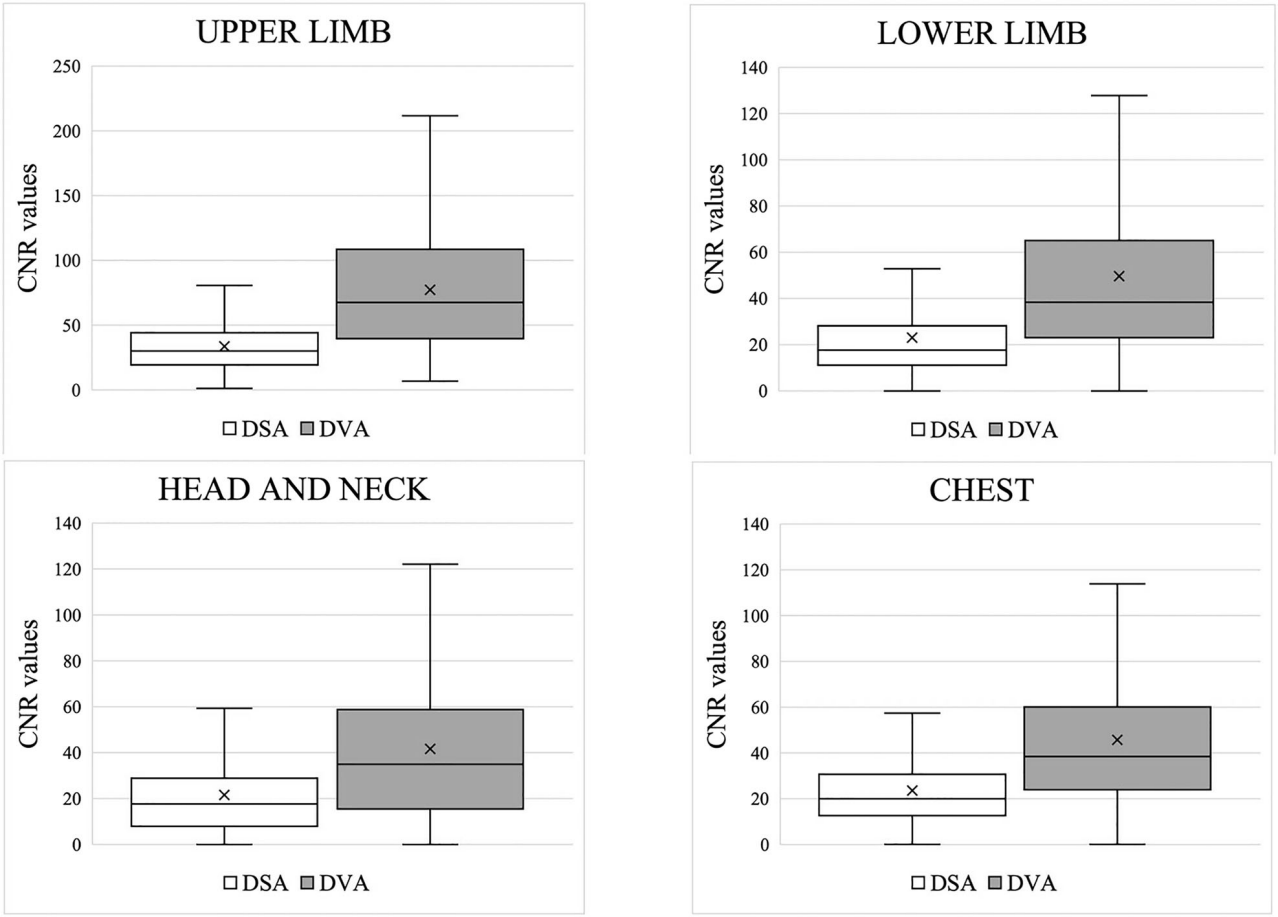
Results of the contrast-to-noise ratio measurements. CNR, Contrast-to-noise ratio; DSA, Digital subtraction angiography; DVA, Digital variance angiography. The mean value, median value, interquartile range, and minimum and maximum values are shown (in symbol form) for each graph. All *p*  <  0.001

The DSA images from which the DVA images were generated contained an average of 15 frames (range, 5–53 frames) per image. A total of 132 DSA-DVA image pairs were evaluated (upper limb, *n* = 14; lower limb, *n* = 56; head and neck region, *n* = 23; and chest, *n* = 39). A total of 3,318 ROIs were selected for the 132 DSA-DVA image pairs (upper limb, *n* = 501; lower limb, *n* = 1,659; head and neck region, *n* = 472; and chest, *n* = 686). The CNR values of the DVA images were found to be significantly higher than those of the DSA images (all *p* < 0.001; see Fig. 2 for box plots of CNR and Table 1). The highest ratio of the CNR of DVA to that of DSA was observed in upper limb AVMs (2.23 (interquartile range 1.18–4.19); Table 1).

**Table 1:** Results of the contrast-to-noise ratio measurements

AVM	DSA CNR, median (IQR)	DVA CNR, median (IQR)	Number of measurements	*p*-value	CNR_DVA_/CNR_DSA_, median (IQR)
All	19.71 (2.52–61.27)	41.29 (1.90–137.02)	3,318	< 0.001	2.00 (0.74–4.49)
Upper limb	29.98 (9.51–72.51)	67.41 (19.90–162.19)	501	< 0.001	2.23 (1.18–4.19)
Lower limb	17.64 (3.54–60.93)	38.32 (4.63–129.24)	1,659	< 0.001	2.06 (0.78–4.63)
Head and neck	17.65 (0.72–57.40)	34.99 (0.30–109.68)	472	< 0.001	1.72 (0.33–4.33)
Chest	20.01 (5.31–53.30)	38.41 (5.11–107.78)	686	< 0.001	1.84 (0.78–4.41)

*AVM* Arteriovenous malformation, *CNR* Contrast-to-noise ratio, *DSA* Digital subtraction angiography, *DVA* Digital variance angiography, *IQR* Interquartile range

### Image analysis: quality assessment

**Fig. 3:**
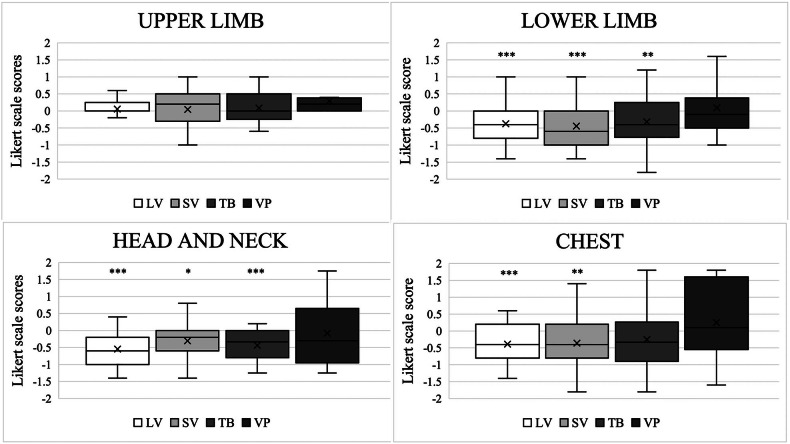
Likert scale comparison of digital subtraction angiography and digital variance angiography images. The mean value, median value, interquartile range, and minimum and maximum values are shown (in symbol form) for each graph. Negative values indicate an advantage of digital subtraction angiography. AVM, Arteriovenous malformation; LV, Large vessel; SV, Small vessel; TB, Tissue blush; VP, Venous phase. * *p* < 0.05; ** *p* < 0.01; *** *p* < 0.001

As illustrated in Fig. 3 and Table 2, the Likert scale results depended on the localization of AVMs. For upper limb AVMs, the visibility of large vessels, small vessels, tissue blush, and the venous phase did not differ significantly between DSA and DVA images. Conversely, DSA images significantly outperformed DVA images in displaying large and small vessels and tissue blush for lower limb and head and neck AVMs, as well as large and small vessels for chest AVMs (see Fig. 3 for box plots of Likert scores and Table 2).

**Table 2:** Likert scale comparison scores for digital subtraction angiography and digital variance angiography images

AVM location	Large vessels	Small vessels	Tissue blush	Venous phase
	Likert score, mean ± SEM (number of comparisons, *n*)			
Upper limb	0.06 ± 0.09 (14)	0.04 ± 0.19 (14)	0.09 ± 0.18 (8)	-0.29 ± 0.14 (8)
Lower limb	-0.38 ± 0.07^***^ (56)	-0.44 ± 0.09^***^ (56)	-0.32 ± 0.12^**^ (49)	0.09 ± 0.21 (16)
Head and neck	-0.55 ± 0.11^***^ (23)	-0.30 ± 0.12^*^ (23)	-0.44 ± 0.11^***^ (19)	-0.08 ± 0.36 (8)
Chest	-0.39 ± 0.09^***^ (39)	-0.36 ± 0.12^**^ (39)	-0.25 ± 0.19 (21)	0.25 ± 0.36 (10)

Negative values represent an advantage of digital subtraction angiography

*AVM* Arteriovenous malformation, *SEM* Standard error of the mean

* *p* < 0.05; ** *p* < 0.01; *** *p* < 0.001

When evaluating the large and small vessels across all regions, Kendall’s *W* coefficient ranged from 0.3 to 0.6, suggesting moderate interrater agreement. A weaker agreement was observed among the raters for the tissue blush and venous phase, with Kendall’s *W* values ranging from 0.1 to 0.3 (Table 3).

**Table 3:** Interrater agreement

Arteriovenous malformation	Kendall’s concordance coefficient *W*	*p*-value
All		
Large vessels	0.368	< 0.001
Small vessels	0.317	< 0.001
Tissue blush	0.288	< 0.001
Venous phase	0.200	< 0.001
Upper limb		
Large vessels	0.463	< 0.001
Small vessels	0.387	< 0.001
Tissue blush	0.402	< 0.010
Venous phase	0.561	< 0.001
Lower limb		
Large vessels	0.364	< 0.001
Small vessels	0.339	< 0.001
Tissue blush	0.312	< 0.001
Venous phase	0.216	< 0.001
Head and neck		
Large vessels	0.423	< 0.001
Small vessels	0.421	< 0.001
Tissue blush	0.359	< 0.001
Venous phase	0.182	< 0.050
Chest		
Large vessels	0.363	< 0.001
Small vessels	0.303	< 0.001
Tissue blush	0.204	< 0.001
Venous phase	0.145	0.108

## Discussion

This study aimed to compare the performance of DVA technology with that of DSA in pediatric patients with AVM undergoing endovascular procedures. The study found that, while DVA exhibited a marked advantage in terms of CNR (CNR_DVA_/CNR_DSA_ ratio of 2.00), it was unable to surpass DSA in terms of visual assessment per the prevailing protocol because the visual image quality of DVA was either equivalent to or marginally inferior to that of DSA. We believe that this small discrepancy in visual quality is of negligible clinical relevance and is unlikely to impact the overall diagnostic efficacy of DVA.

Regarding CNR, the present results agree with previous studies on vascular interventions. In these studies, DVA consistently demonstrated a higher CNR than DSA. Most previous studies focused on lower limb endovascular procedures, with a median overall CNR_DVA_/CNR_DSA_ ratio between 1.84 and 2.8 [12, 13, 18, 19, 21]. These results align closely with our findings (median overall CNR_DVA_/CNR_DSA_ ratio of 2.00), particularly when considering the results in the lower (CNR_DVA_/CNR_DSA_ ratio of 2.06) and upper limb regions (CNR_DVA_/CNR_DSA_ ratio of 2.23), where radiation must penetrate less tissue, and motion-related artifacts affecting image quality are minimal. Studies in the carotid region have reported CNR ratios ranging from 2.06 to 2.25, with DVA prevailing [17]. The rapid and irregular breathing patterns observed in children, in conjunction with involuntary swallowing or crying, have been shown to induce significant motion in the head and neck region, thereby contributing to motion artifacts during imaging procedures. This may explain why lower CNR values were noted in cases of pediatric AVMs located in the head and neck region, with a median CNR_DVA_/CNR_DSA_ ratio of 1.72. In the context of transarterial chemoembolization of the liver, an intervention susceptible to motion artifacts due to respiration and cardiac pulsations, the median overall CNR_DVA_/CNR_DSA_ ratio was 1.24 [15]. Notably, our study revealed higher CNR values in the chest region (CNR_DVA_/CNR_DSA_ ratio of 1.84), where comparable motion artifacts, although present, were less pronounced than in the upper abdominal region. The interference of bowel movements and intestinal gas, which can impede the interpretation of images during transarterial chemoembolization of the liver, may explain the difference between the thoracic and abdominal regions. It is also noteworthy that the AVMs observed in this study were in the chest wall, rather than in the lungs. The findings of a recent intervention, prostatic artery embolization, are very promising, evidencing a more than fourfold advantage of DVA (CNR_DVA_/CNR_DSA_ ratio of 4.11) [14].

In contrast to prior studies, which indicated the superiority of DVA in visual quality assessments, this study showed that DVA was not superior to DSA in visual quality. The following factors may be responsible for this discrepancy. First, pediatric patients have smaller vessels and anatomical structures that are sensitive to even the slightest movements, and motion-related artifacts may be amplified in certain instances by the variance-based DVA algorithm. Second, (super)selective angiography typically produces high-quality DSA images, thereby setting a high standard that is challenging to exceed. Third, children’s smaller body size and lower tissue mass facilitate higher image quality with conventional DSA techniques. Given the optimization of DSA for high-quality imaging, a “ceiling effect” may emerge, where further enhancements in visual quality become difficult to achieve.

The improved CNR observed with DVA suggests a possible quality reserve that could be employed for dose management in pediatric patients, who are more vulnerable to the long-term consequences of radiation. In a prospective study conducted in 2021, Gyánó et al. found that DVA allows for an approximately 70% reduction in DSA-related radiation exposure in lower extremity interventions [18]. The results of a recently published randomized clinical trial demonstrated that the quality reserve of DVA established in previous retrospective studies can be used in selective lower limb procedures to reduce radiation exposure in clinical practice without compromising image quality or the diagnostic value of angiograms [19].

Our study has several limitations. First, the sample size is small (10 patients; 132 image pairs). Second, the study design is retrospective and observational, which introduces potential selection bias, as only patients who underwent clinically indicated procedures for AVM treatment at a single center were included. Third, image interpretation was influenced by subjective expert judgment, although interrater agreement was moderate to strong for most parameters. These factors may limit the generalizability of the findings.

Prospective studies are needed to validate the purported benefits of DVA in pediatric radiological interventions, especially regarding radiation dose reduction. These studies can intentionally reduce the volume of contrast agent and radiation exposure during DVA acquisitions to test whether image quality remains diagnostically acceptable, and then use stepwise dose reduction tiers to set safety thresholds for each vascular region. It is essential to adapt imaging protocols to the distinctive physiological characteristics of children, including implementing age-appropriate sedation strategies to minimize motion during acquisitions, adjusting frame rates, and utilizing shorter acquisition windows. Additionally, the optimization of motion correction algorithms is crucial. Establishing standardized pediatric DVA protocols that incorporate these adaptations would support safer imaging practices.

In conclusion, the results of this study propose that DVA possesses a considerable capacity for enhancing CNR. In light of the encouraging outcomes revealed in earlier prospective studies conducted on lower extremity endovascular procedures, our findings offer a promising avenue for addressing the critical issue of radiation dose management, particularly in the context of pediatric populations. Consequently, further exploration is warranted to investigate the potential of DVA to reduce radiation exposure while maintaining diagnostic image quality in pediatric patients.

## Data Availability

The data presented in this study are available upon request from the corresponding author. Due to concerns regarding patient privacy, the data are not publicly available.

## Abbreviations

AVM: Arteriovenous malformation
CNR: Contrast-to-noise ratio
DSA: Digital subtraction angiography
DVA: Digital variance angiography
ROI: Region of interest
